# “Elastic Stretch Cavity Building” System in Endoscopic Thyroidectomy of Giant Thyroid Tumors

**DOI:** 10.3389/fonc.2022.871594

**Published:** 2022-05-27

**Authors:** Gaoxiang Chen, Hai Zhang, Cheng Li, Limu Wen, Jianan Zhang, Minhua Wu, Weifeng Teng, Xiaochun Ji, Yong Luo, Weizhu Wu

**Affiliations:** Department of Thyroid and Breast Surgery, Ningbo Medical Center Lihuili Hospital, Ningbo, China

**Keywords:** mixed cavity building, endoscopy, large thyroid masses, resection, cavity builder

## Abstract

**Objective:**

To analyze the clinical characteristics of patients with large thyroid tumors underwent endoscopic thyroidectomy using the “elastic stretch cavity builder” system.

**Methods:**

This retrospective case series study included thyroid tumor patients admitted to the Ningbo Medical Center Li Hui li Hospital between September 2017 and November 2021. The self-developed “elastic stretch cavity builder” was used to elastically lift the anterior cervical flap, combined with low-pressure (3 mmHg) high-flow CO2 inflation, and create a working cavity for endoscopic thyroidectomy.

**Results:**

This study included 13 patients for analysis. The endoscopic thyroidectomy duration was 92-170 min (mean, 123 ± 24min). The maximum transverse plane diameter of the glands was 5.0-6.2 cm (mean, 5.3 ± 0.3 cm). The maximum sagittal plane diameter was 6.8-10.0 cm (mean, 7.6 ± 0.9 cm). After the “elastic stretch cavity builder” lifted the cervical flap, the height of the subcutaneous region was increased by 1.3 ± 0.2cm without affecting cervical activity. There was no residual scar in the anterior cervical skin puncture hole. All patients were satisfied with the cosmetic with the cosmetic satisfaction score was 3.4 ± 0.5.

**Conclusion:**

The novel mixed cavity building model established by the “elastic stretch cavity builder” might provide the surgeon with additional longitudinal cervical operating space while improving the stability of the space and saving human effort.

## Introduction

Thyroid nodules are found in up to 50%-65% of healthy individuals (1, 2). The lifetime risk of having a thyroid nodule is 10% (3). Most thyroid nodules are asymptomatic. Palpable nodules often are discovered on physical examinations, and nonpalpable nodules frequently are detected incidentally on imaging studies performed for unrelated reasons. Thyroid nodules may be caused by both benign (about 90%) and malignant (about 10%) lesions (1, 2). While thyroid nodules may be associated with thyroid dysfunction or local mass effects, the primary clinical concern is to identify and treat lesions that are malignant or at high risk for malignancy (1, 4).

However, many patients have high cervical cosmetic requirements, and many are eligible for endoscopic thyroidectomy (5). Given its excellent cosmetic outcomes and some minimally invasive features, it integrates therapeutic and cosmetic effects and has been gradually favored by doctors and patients, giving rise to oncoplastic thyroidectomy (6). Unlike laparoscopy, there is no natural cavity in the neck, and the skin flap needs to be separated during the procedure to establish a cervical working cavity. Although the large-area skin flap separation can increase the volume of the working cavity to a certain extent and reduce the difficulty of the procedure, the additional trauma caused to the patient cannot be ignored (5). Therefore, the authors consider that the separation of the skin flaps should adhere to the principle of the smallest effective area, but the smaller the space, the more important its stability. Conventional endoscopic thyroidectomy needs to fill the subcutaneous space with CO2 (6-8 mmHg) and uses air pressure to establish and maintain the operating space. However, the cervical operating space is limited and has poor stability due to the strong fluidity.

The authors developed the “elastic stretch cavity builder” (patents # ZL 2015 2 0962366.7 and ZL 2016 2 0599586.2). The elastic stretch cavity builder mainly includes the following components: 1) Self-developed and designed special right-angle hooks with different specifications and sizes: ‘S’ type hooks for lifting neck flaps and ‘U’ type hooks for pulling muscles and trachea. The new hook can be applied to different surgical approaches and usage scenarios. Such as hanging neck flap, pull neck muscles, trachea, etc. 2) Hook displacement regulator: The regulator adopts the way of thread adjustment to realize the continuous stepless adjustment of the hook. The regulator connects the spring and the hook to achieve elastic connection rather than hard traction. The regulator can be fixed at any bracket position through the buckle. 3) Arc track bracket: the bracket adopts a symmetrical two-stage structure, and the buckle firmly connects the middle to form an arch-shaped track bracket, which is connected with the slide rails on both sides of the surgical bed through the L-shaped connection components. The arc stent has two orbital forms, and the patient is in the upper position with a rectangular cross-section, which is mainly used to carry the hook of the vertical lifting flap; the track on both sides of the patient adopts a cross-sectional hexagonal track shape, which can be adjusted according to the surgical needs (**Supplementary Figure 1**).

From September 2017 to November 2021, the authors developed the “elastic stretch cavity builder” to complete endoscopic thyroidectomy successfully in 156 patients (7), including 13 with a maximum diameter of thyroid of ≥5 cm in the transverse plane. Therefore, this study aimed to analyze the clinical characteristics of patients with large thyroid tumors underwent endoscopic thyroidectomy using the “elastic stretch cavity builder” system. The procedures were performed mainly by three surgeons (Chen GX, Zhang H, Li C), all of whom have more than 5 years of experience in laparoscopic thyroid surgery prior to initiation of the current study.

## Methods

### Study Design and Patients

This retrospective case series study included thyroid tumor patients admitted to the Ningbo Medical Center Li Hui li Hospital between September 2017 and November 2021. The present study was approved by the Ethics Committee of the Ningbo Medical Center Li Hui li Eastern Hospital (No. DYLL2017009). Since this study was a retrospective study, the informed consent form was exempted.

Except for cystic tumors, all patients underwent preoperative fine-needle aspiration biopsy and intraoperative rapid pathological section. Before surgery, cervical color Doppler ultrasonography and CT scan were performed to assess the location and size of the tumor. The inclusion criteria were 1) confirmed thyroid tumor and underwent endoscopic thyroidectomy, 2) undergoing thyroidectomy for the first time, 3) the maximum diameter of the gland in the transverse plane (D) was ≥ 5 cm, and 4) the lower boundary of the gland was located above the thoracic inlet plane when the neck was in the hypsokinesis and hyperextension position. The exclusion criteria were 1) severe cardiopulmonary dysfunction, chest wall anomalies, or other surgical contraindications. 2) malignant thyroid tumors. 3) tumors with a Bethesda System for Reporting Thyroid Cytology (TBSRTC) classification of category 3 or higher. 4) Retrosternal goiter. 5) Preoperative tracheal compression with obvious dyspnea.

### Data Collection

The clinical data were retrospectively obtained from the hospital medical records, including age, sex, pathological results, the maximum diameter of the glands in the horizontal cross-section, the maximum diameter in the sagittal position, the additional height of the subcutaneous space after the cervical flap was lifted by the elastic stretch cavity builder, surgery time, postoperative negative pressure drainage volume, length of postoperative stay, postoperative complications, and surgical video (**Supplementary Video 1**). Postoperative complications including hypocalcemic convulsions, temporary hoarseness, and skin ecchymosis burns were collected. Patients were asked about their satisfaction with overall cosmetic outcomes, rating from 1 (very unsatisfied), 2 (unsatisfied), 3 (satisfied), to 4 (very satisfied). The first follow-up was performed 1 month after endoscopic thyroidectomy and every 6 months after that. Follow-up was conducted through outpatient revisits and telephone.

### Statistical Analysis

Only descriptive statistics were used. Data were presented as mean ± standard deviation (SD), median (interquartile range), or n (%).

## Results

**Figure 1:**
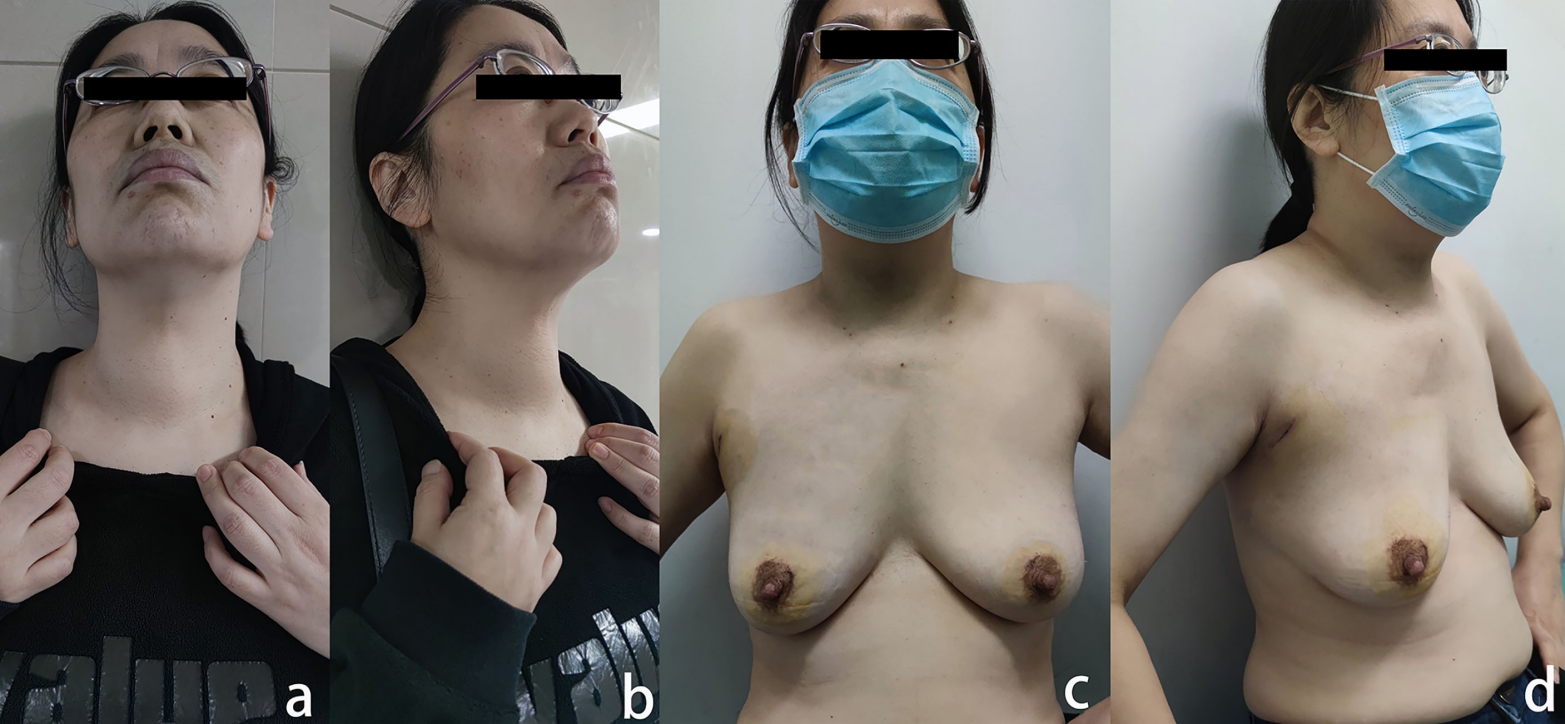
**(A)** Patients’ preoperative frontal appearance; **(B)** patients’ preoperative lateral appearance; **(C)** The front appearance of the patients at the removal 1 week after operation; **(D)** the side appearance of the patients.

A total of 13 patients successfully underwent the endoscopic hemithyroidectomy procedure. Routine pathology revealed 11 nodular goiter and two thyroid adenomas. The maximum diameter of the glands in the transverse plane (D) was 5.0-6.2 cm, with a mean of 5.3 ± 0.3 cm. The maximum diameter in the sagittal plane (S) was 6.8-10.0 cm, with a mean of 7.6 ± 0.9 cm. After the elastic stretch cavity builder lifted the cervical flap, the height of the subcutaneous region was increased by 1.3 ± 0.2 cm. The surgery time was 92-170 min, with a mean of 123 ± 24min. The postoperative negative pressure drainage volume was 125-240 ml, with a mean of 180 ml. Patients were discharged from the hospital after extubation for safety reasons, the mean length of stay was 4.5 days. After surgery, the cervical activity was not affected, and there were no notable complications. One patient had temporary hoarseness but returned to normal at a 1-month follow-up after surgery. Postoperative B-mode ultrasound reexaminations did not reveal tumor recurrence. All patients were satisfied with the cosmetic outcomes with the cosmetic satisfaction score was 3.4 ± 0.5 (**Table 1** and **Figure 1**).

**Table 1:** Baseline features, surgical data, and postoperative data.

Characteristics	Patients (n=13)
Age, mean (years)	34
Sex, n (%)	
Male	4
Female	9
Pathological results, n (%)	
Nodular goiter	11 (84.6)
Thyroid adenoma	2 (15.4)
Surgery-related data	
Maximum diameter of the glands in the horizontal cross-section, cm (range, mean **±** SD)	5.0-6.2 (5.3 ± 0.3)
Maximum diameter in the sagittal position, cm (range, mean **±** SD)	6.8-10.0 (7.6 ± 0.9)
Height of the additional subcutaneous space after the cervical flap was lifted by the elastic stretch cavity builder, cm (range, mean **±** SD)	1.3 ± 0.2
Surgery time, min	92-170 (123 ± 24)
Postoperative negative pressure drainage volume, ml	125-240 (180 ± 38))
Length of stay, days (range, mean **±** SD)	4.5
Postoperative complications	
Hypocalcemic convulsions	0
Temporary hoarseness	1
Skin ecchymosis burns	0
Cosmetic satisfaction score	3.4 ± 0.5

### Case Report

A 42-year-old female patient complained of a thyroid space-occupying mass for 5 years. The patient underwent a physical examination 5 years ago. Thyroid B-ultrasound showed a mass with a clear boundary and regular shape with mixed echo and a size of about 3×4 cm in the right thyroid, which was diagnosed as a right thyroid nodule, TI-RADS 3. Since then, the patient failed to attend follow-up regularly and felt that the neck mass gradually increased. Five days ago, the patient visited the outpatient clinic. The thyroid B-ultrasound showed that the right thyroid nodule was about 10×6.3 cm in diameter. The patient felt no other discomforts. The patient was diagnosed with a thyroid nodule and admitted to the hospital.

Physical examination after admission showed that the patient had a clear mind and acceptable mental state, with a body temperature of 36.8°. The trachea was displaced to the left, and an obvious bump protrusion could be observed in the right neck, about 10×5 cm. The mass was flexible and smooth and could move up and down with swallowing without obvious tenderness.

**Figure 2:**
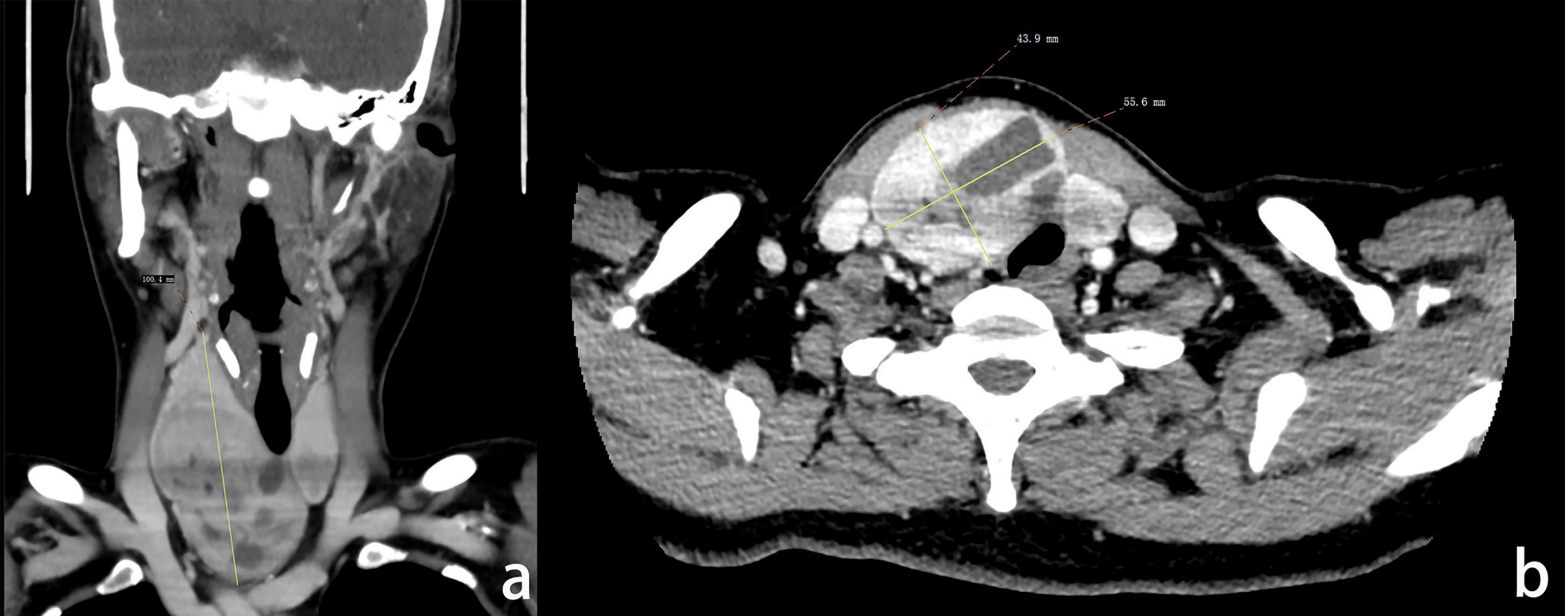
**(A)** The coronal image of enhanced CT of the patient’s neck, the maximum diameter of the gland was 100.4 mm; **(B)** The horizontal image of enhanced CT of the patient’s neck, the maximum diameter of tumor cross-section was 55.6×43.9 mm.

Enhanced computerized tomography of the neck showed enlargement of the right thyroid gland lobe, multiple cystic low-density shadows, calcifications, and narrowing of the trachea. The diagnosis was a right thyroid mass with calcification (**Figure 2**). The final pathological diagnosis was an adenomatous nodule of the right thyroid, with a maximum diameter of 10 cm, and focal capsule thickening with fibrosis and calcification.

### Surgical Procedures

**Figure 3:**
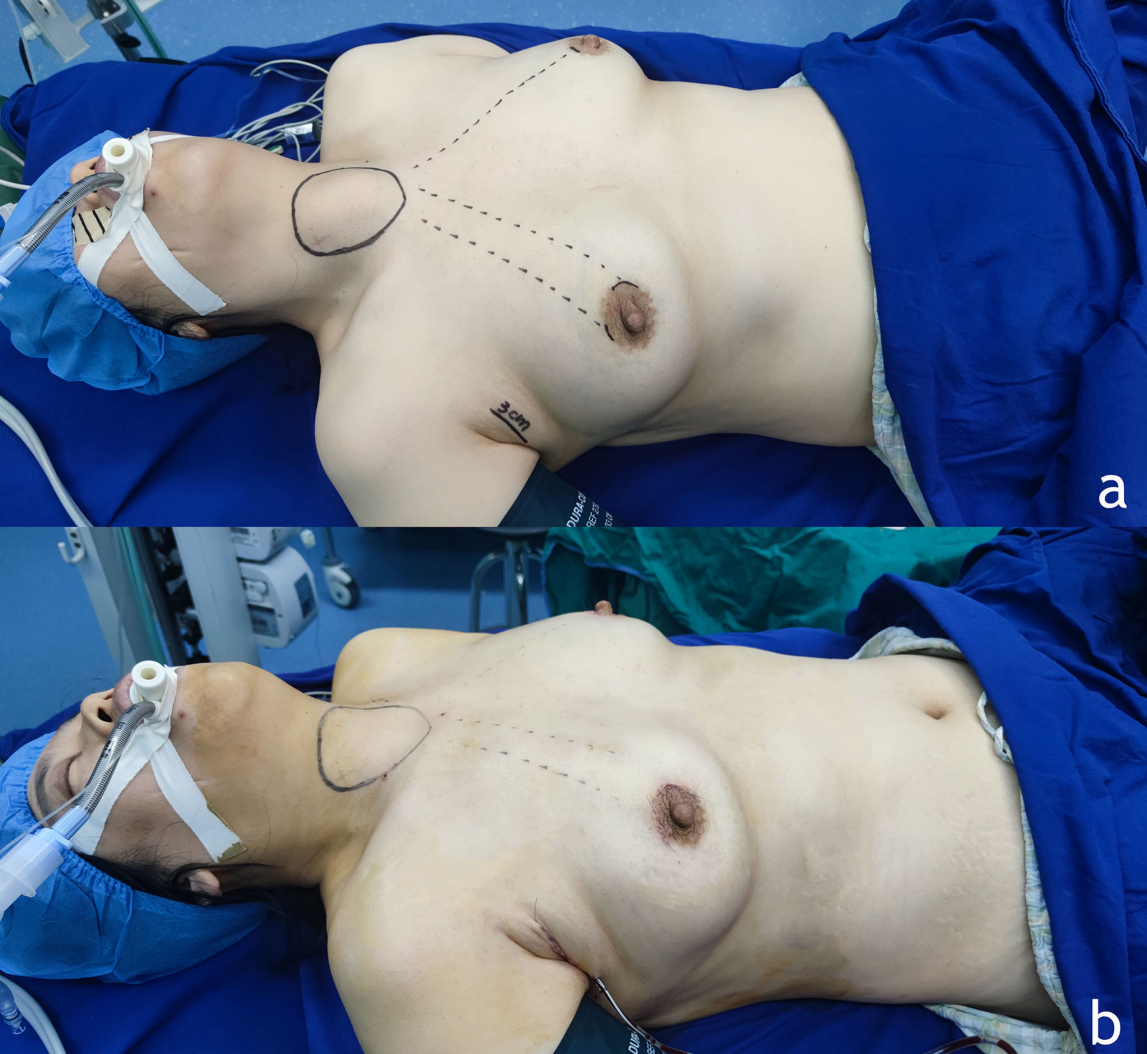
**(A)** Preoperative position and incision position. The female patients adopted the full-areola incision, and the arc-shaped areola incision with a length of about 1.5 cm was made in the 3 - point direction of the right breast as the observation hole. A 0.5 cm incision was made in the direction of left and right areola 10∽11 points as the operating hole. At the level of the front line of the right armpit of the patient, the wrinkled incision with a length of about 3 cm was taken, and the specimen was taken out completely from the armpit incision after packing. **(B)** Appearance after placement of drainage tube.

General anesthesia was performed with tracheal intubation. The patient was in the supine position with head high and feet low, legs apart, and shoulders raised high. The neck was in the hypsokinesis and hyperextension positions. In female patients, the full-areola incision was adopted, and the arc-shaped areola incision with a length of about 1.5 cm was made in the 3-point direction of the right breast as the observation port. A 0.5-cm incision was made in the direction of the left and right areola as the operating port. A trocar was placed, and CO2 was injected to maintain pressure at 6 mmHg (**Figure 3**). After inserting the endoscope, an ultrasonic knife or an electric knife was used to separate the subcutaneous loose connective tissues to establish an operating space up to the upper edge of the thyroid cartilage and bilaterally to the midline of the sternocleidomastoid muscle.

**Figure 4:**
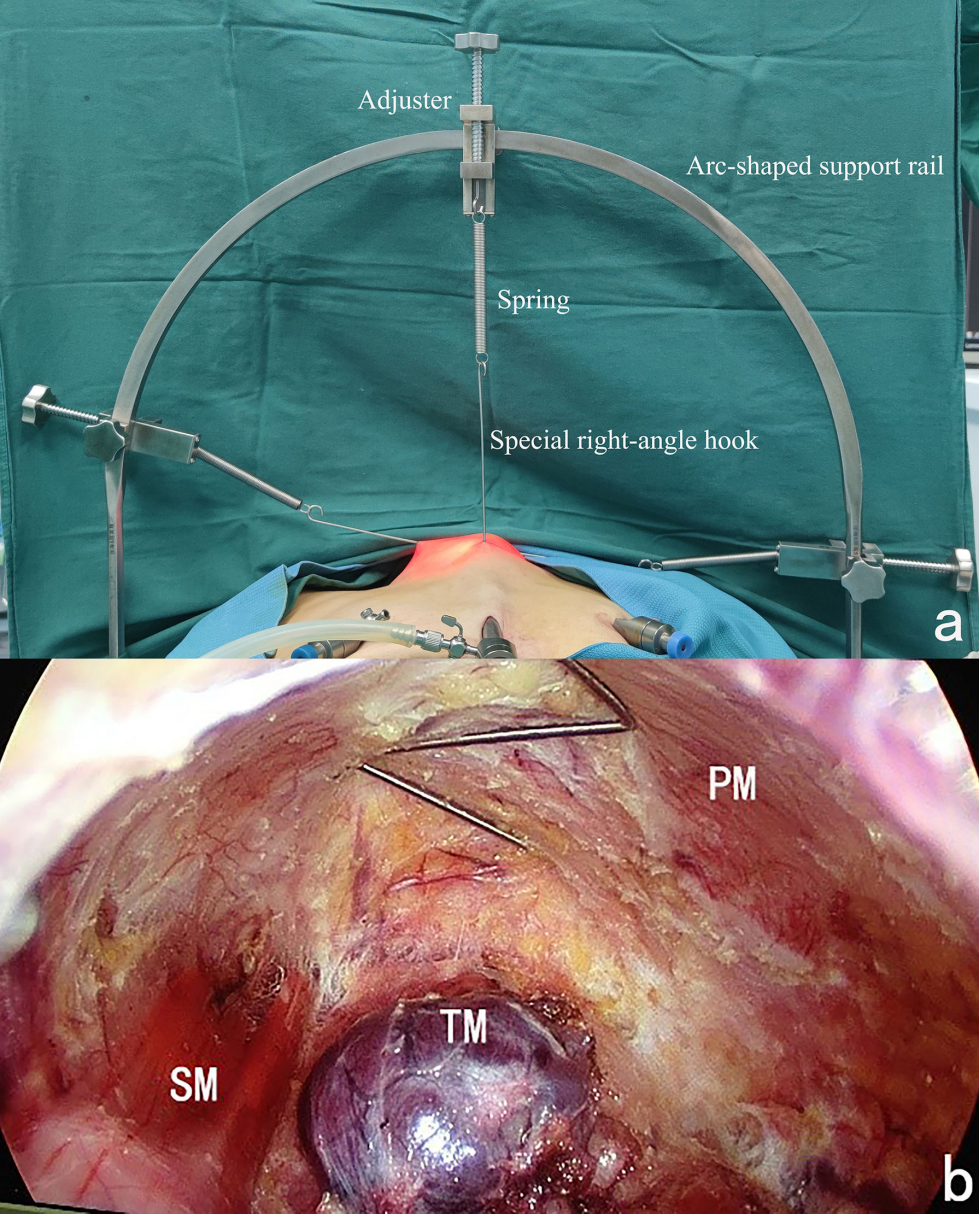
**(A)** Outer schematic diagram of the use of the “elastic stretch cavity builder”. **(B)** Inner schematic diagram of the use of the “elastic stretch cavity builder”. PM, Platysma myoides; SM, Sternocleidomastoid muscle; TM, Thyroid tumor.

The “elastic stretch cavity builder” was used. At the level of the suprasternal fossa, the special right-angle hook was inserted into the skin and placed on the deep surface of the cervical flap. The cervical flap was lifted vertically upwards. The hook was connected to the adjuster by a spring and fixed on the arc-shaped support rail. The carrier height knob was rotated until the flap was just tight. The CO2 pressure was reduced to 0-3 mmHg, and the thyroid lobectomy was completed. During lobectomy, multiple sets of special right-angle hooks could be used to reversely pull the strap muscles and trachea to establish the operating cavity space (**Figure 4**).

The isthmus was separated along the pretracheal space. The inferior pole of the gland was pulled to the head to dissociate, condense, and close the inferior pole vein and the lowermost blood vessel. Flipping and lifting the gland were performed to the opposite side to dissociate the lateral border of the gland from bottom to top. The middle thyroid veins were dissociated, and the inferior thyroid artery was sought. Using the inferior thyroid artery and its branches as a mark, the recurrent laryngeal nerve (RLN) was sought, and the RLN was exposed from bottom to top to the vicinity of the inlet point. The ligament of Berry was dissociated. The dorsal side of the gland was dissociated upwards across the inlet point, leaving the upper parathyroid in situ. Then, the upper pole vessel cap was condensed and closed to complete the thyroidectomy.

**Figure 5:**
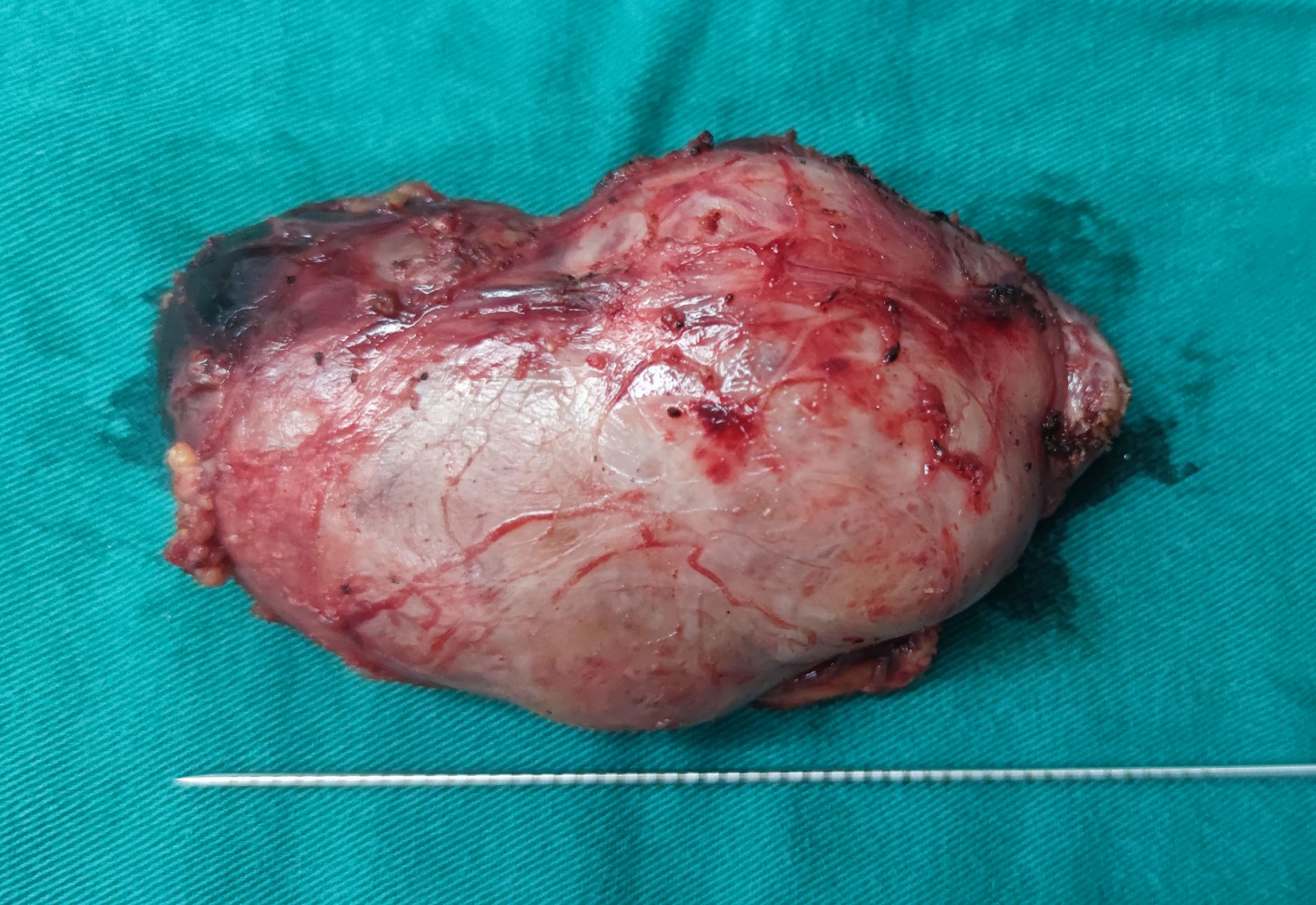
The completely removed gross specimen of the right gland tumor of the patient (the distance between the scales was 2 mm in the figure).

At the anterior level of the right armpit of the patient, the wrinkled skin incision with a length of about 3 cm was taken, and the flap was separated from the surgical cavity inward and upward along the surface gap of the pectoralis major myofascial under direct vision. After packing, the specimen was completely removed from the axillary incision (**Figure 5**).

**Figure 6:**
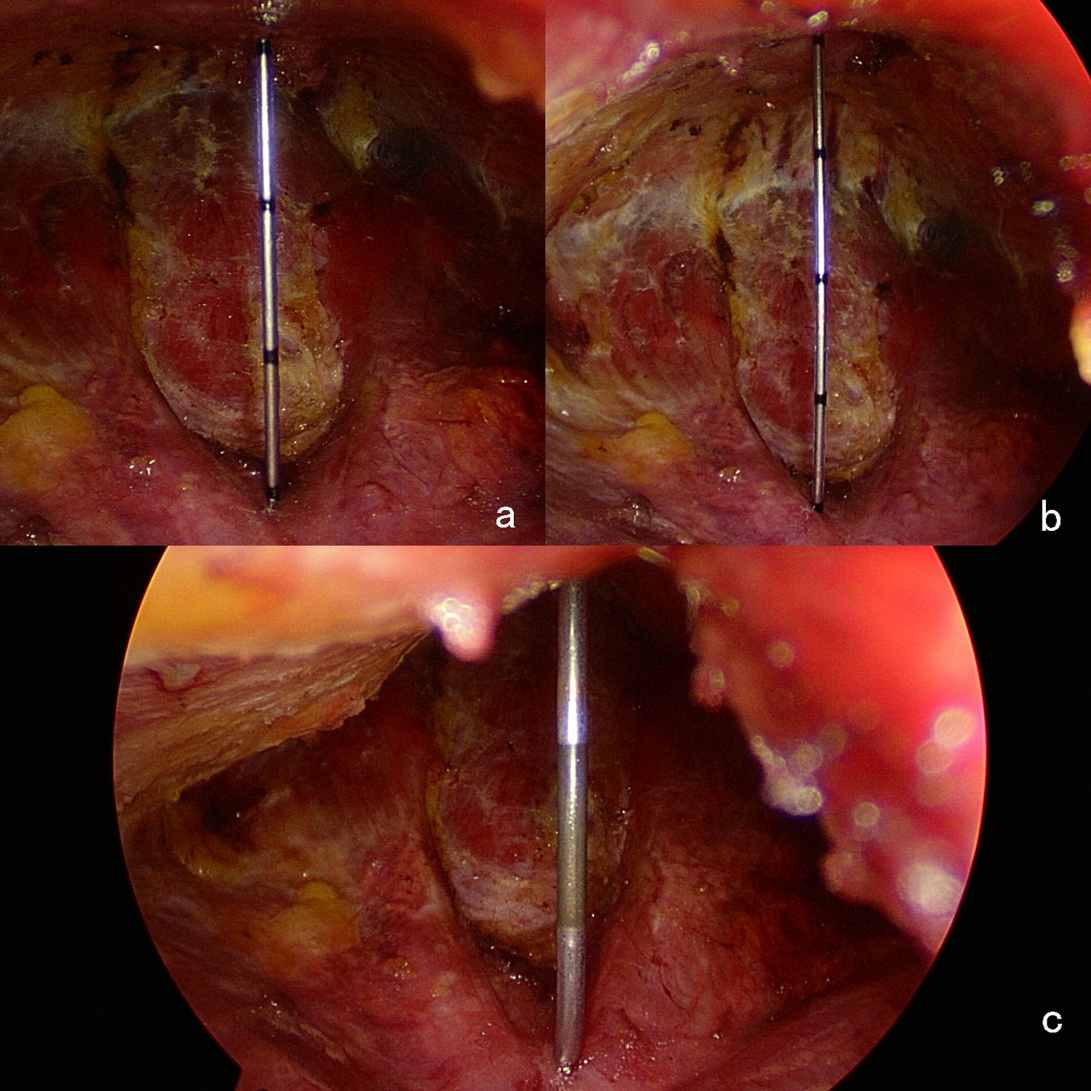
**(A)** Measuring the height of the subcutaneous space before the elastic suspension. **(B)** The height of the subcutaneous space is increased by about 1 cm after elastic suspension. **(C)** There is still sufficient field of view and operating space under vacuum aspiration. When the vacuum aspiration is performed during the procedure, the spring component of the elastic lifting cavity building system is stretched and extended. The hook and the skin flap are compressed downward to adapt to the negative pressure in the cavity. At this time, the cavity space is not completely collapsed, and it can maintain a sufficient field of view and operating space. When the vacuum aspiration ends, the spring component quickly shrinks and retracts to restore the space to its initial state.

After completing the surgery, the subcutaneous measuring needle was inserted vertically from the highest point of the flap to measure the vertical space height (h) from the highest point of the flap to the tracheoesophageal groove under conventional CO2 inflation and the vertical space height (H) from the highest point of the flap to the tracheoesophageal groove after the elastic stretch cavity builder lifted the flap (**Figure 6**).

The patient was discharged after extubation when the drainage volume was less than 20ml a day after operation. The suture was removed 1 week after the operation, and the cosmetic neck score of the patient was 3 points. The replacement therapy with levothyroxine 50 μg qm po was administrated after the operation. Thyroid function was normal 1 month after the operation.

## Discussion

The mechanical cavity building method provides a better spatial stability than that of the traditional CO2 insufflation cavity building method. The working chamber can be kept stable and the surgical field can be kept clear with the help of mechanical lifting and continuous intraoperative negative pressure suction. Many scholars have applied the mechanical cavity building method in gasless transaxillary endoscopic thyroidectomy and achieved ideal results (8–10).

Since 2017, our clinical center has begun to perform ipsilateral thyroidectomy by using the gasless transaxillary approach. We found the space built by the special hook was stable and the view during the surgery was always very clear due to the absence of water mist. However, during the surgery, the surgeon needs to establish a working space passing through the sternocleidomastoid muscle, which results in a very restricted operative space for tumors with a short diameter larger than 5cm, so we usually apply this approach in the resection of early differentiated thyroid cancer and benign thyroid tumor with a diameter less than 5cm.

From 2017, our clinical center has developed a simple elastic stretch cavity builder and applied it in endoscopic thyroid surgery *via* the breast approach. During these years, our team has gradually improved the cavity builder and finally achieved the current structure.

The present study suggested that the novel mixed cavity building model established by the “elastic stretch cavity builder” might provide the surgeon with additional longitudinal cervical operating space and improve the stability of the space, thus making it possible for endoscopic thyroidectomy of large thyroid masses that would be difficult to remove endoscopically.

Previous studies of endoscopic thyroidectomy reported relatively good outcomes (5, 11–13). Endoscopy depends on the operating space, but there is no natural cavity space in the neck (5). Therefore, the present study adopted the two-step mixed cavity building method of “inflation for cavity building, lifting and maintaining” to establish the space. Mechanical lifting for cavity building alone will make the subcutaneous space not broad enough. Moreover, it is easy to cause iatrogenic injuries, making the cavity building very difficult and complicated. Selecting the conventional CO2 inflation (pressure of 6 mmHg) can make the loose connective tissue to dilate and expand evenly. As a result, dissociating the skin flap would be easier. Nevertheless, after the separation of the subcutaneous space is completed, maintaining the stability of the space was even more important. The present study used the patented “elastic stretch cavity builder” to lift the skin flap vertically to form a “landing” type operating space on the neck to establish a mixed cavity building model with mechanical cavity building as the main one and inflatable cavity building (CO2 pressure of 0-3 mmHg) as the auxiliary one. Then, CO2 perfusion was no longer necessary for space maintenance. During intraoperative conditions such as bleeding and continuous vacuum aspiration, the spring components of the “elastic stretch cavity builder” can stretch freely to adapt to the changes in pressure in the cavity, and the operating space will not collapse. As a result, a stable and continuous field of view can be obtained during surgery to ensure the accuracy and safety of the procedure.

Prior work had tried to completely turn off the CO2 inflation during the procedure and depend on mechanical lifting alone to maintain the operating space. However, due to the relatively small cervical space, the smoke caused by the ultrasonic knife or electrocoagulation hook is difficult to dissipate, which affects the procedure. The mixed cavity building model by elastic lifting and low pressure (CO2 pressure of 3 mmHg) high flow rate (20 L/min) can form continuous gas exchange in the surgical cavity and quickly remove the smoke caused by the ultrasonic or electric procedures. Moreover, due to the decrease in inflation pressure, the likelihood of complications caused by CO2 perfusion is also reduced, and surgical safety is further ensured.

Furthermore, during thyroid lobectomy, the “elastic stretch cavity builder” can be used to concurrently bear multiple sets of hooks to oppositely pull bilateral strap muscles or sidewalls of the trachea, to replace the manual hooks to establish a stable operating cavity space. Hence, this system can be used to assist surgery and improve the surgical fields in other ways than simply lifting the skin.

After intraoperative observation and repeated review of the surgical videos after surgery, the following observations can be made. For those patients whose lower boundary of the gland is located above the thoracic inlet plane after the neck is in the hypsokinesis and hyperextension position (namely, the gland moves up to the neck in the hypsokinesis position), the maximum diameter in the sagittal plane of the gland has little impact on the procedure. Conversely, glands with a large diameter in the transverse plane cause serious problems for the surgeon during the procedure due to the limited operating space and difficulty in exposure. During the sequential lobectomy, the ligament of Berry is the densest connection between the gland and the surrounding tissues. It is located deep in the capsule behind the gland and adjacent to the RLN inlet point, so safely and smoothly releasing the ligament of Berry near the nerve inlet point often determines the success of the surgical procedure. The surgeon can lift and flip the gland to the opposite side to expose the anatomical structure of the dorsal side of the gland, thereby making it possible for thyroid lobectomy. But for patients with large thyroid masses, the deep space of the glands is limited, and it is very difficult for exposure and procedure. Therefore, total endoscopic thyroidectomy of large thyroid masses roughly indicates full exposure of the anatomical structure near the RLN inlet point during the procedure for observation and operation by the surgeon. Lifting the cervical flap by the “elastic stretch cavity builder” can significantly increase the longitudinal operating space in the cavity (an increase of longitudinal height by 1.3 ± 0.3 cm). Thus, it can provide extra longitudinal operating space for the surgeon while improving the space stability, which is crucial for the limited cervical space. Hence, it facilitates flipping, lifting, and other procedures of the glands for the surgeon. In addition, the wide field of view and broad operating space can reduce the difficulty and improve the procedure’s safety, especially when the dorsal capsule is dissected with high requirements for operation accuracy.

**Figure 7:**
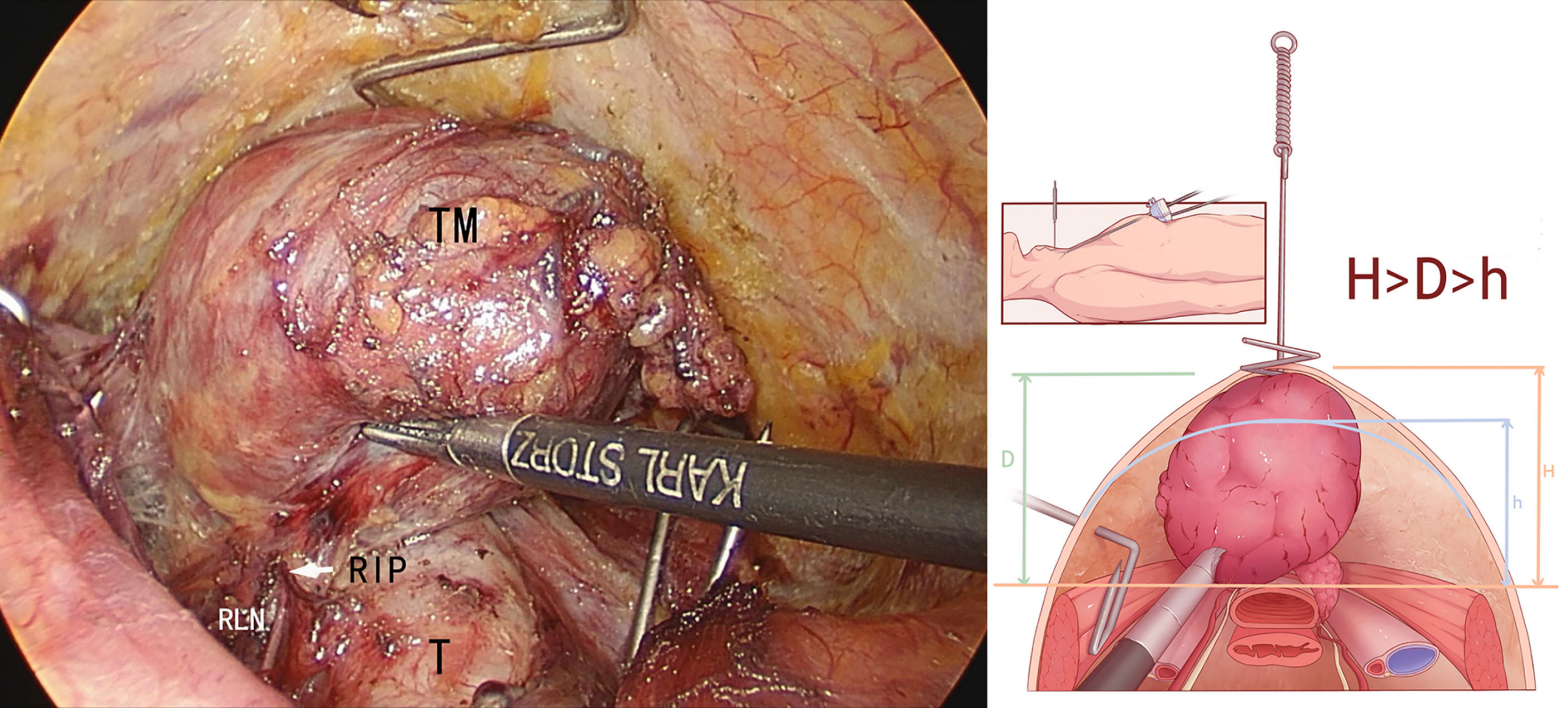
Before the “elastic stretch cavity builder” lifts the skin flap, tumor diameter (D) was larger than the available height (h) (D>h), and the tumor could not be fully flipped to the opposite side. After the “elastic stretch cavity builder” is used to lift the skin flap, the expanded height (H) is larger than D and h (H>D>h), and the recurrent laryngeal nerve (RLN) inlet point can be fully exposed, making it possible for tumor resection. TM, thyroid tumor; T, trachea; RLN, recurrent laryngeal nerve; RIP, RLN inlet point.

Therefore, the following findings can be obtained. Compared with the maximum diameter in the sagittal plane (S) of the gland, the maximum diameter in the transverse plane (D) is the key determinant for endoscopic thyroidectomy. The dissociation of the ligament of Berry near the RLN inlet point is a key step in the procedure. Whether the area can be fully exposed for observation and operation by surgeons is the key to the procedure’s success. The lower boundary of the gland is above the plane of the tracheoesophageal groove by lifting and flipping so that the ligament of Berry near the nerve inlet point can be fully exposed, and the gland can be removed safely and smoothly. By elastic traction, the vertical height from the highest point of the flap to the tracheoesophageal groove can be increased from h to H and greater than the maximum diameter of the gland in the transverse plane D so that the resection of large thyroid tumors can be successfully performed (**Figure 7**).

Although the novel mixed cavity construction model might expand the surgical indications, there are still some contraindications, such as locally advanced thyroid malignancy, retrosternal goiter, preoperative tracheal compression with obvious dyspnea, and special types of thyroid cancer, such as medullary thyroid carcinoma and anaplastic thyroid carcinoma.

This study had several limitations. It was a retrospective study of patients from a single hospital. Hence, the sample size was small. As the “elastic stretch cavity builder” is patented but not commercialized yet, future studies will rely on the demonstration of the advantages of the system to convince other surgeons of using it and increasing the sample size. Randomized controlled trials needed to be planned in the future.

In summary, the novel mixed cavity building model established by the “elastic stretch cavity builder” can save human resources, provide the surgeon with additional longitudinal cervical operating space and improve the stability of the space, thus the study might provide a new treatment for endoscopic thyroidectomy for large thyroid masses.

## Data Availability Statement

The raw data supporting the conclusions of this article will be made available by the authors, without undue reservation.

## Ethics Statement

The studies involving human participants were reviewed and approved by Ningbo Medical Center Li Hui li Eastern Hospital (No. DYLL2017009). Written informed consent for participation was not required for this study in accordance with the national legislation and the institutional requirements. Written informed consent was obtained from the individual(s) for the publication of any identifiable images or data included in this article.

## Author Contributions

GC provided the conceptualization of this study, drafted the manuscript, and performed data analysis. HZ contributed to the study’s design and collection of data. CL, LW, JZ, MW, and WT worked on investigation and data collection. XJ, YL, and WW conducted the critical revision of the manuscript. All authors contributed to the article and approved the submitted version.

## Funding

This work was supported by the Science and Technology Program for Public Wellbeing of Ningbo (2017C50067).

## Conflict of Interest

The authors declare that the research was conducted in the absence of any commercial or financial relationships that could be construed as a potential conflict of interest.

## Publisher’s Note

All claims expressed in this article are solely those of the authors and do not necessarily represent those of their affiliated organizations, or those of the publisher, the editors and the reviewers. Any product that may be evaluated in this article, or claim that may be made by its manufacturer, is not guaranteed or endorsed by the publisher.

## Supplementary Material

Supplementary Figure 1 The components of the “elastic stretch cavity builder”.

Supplementary Video 1 “Elastic stretch cavity building” system in endoscopic thyroidectomy of giant thyroid tumors.
