# Supplementary figures and images for: “Elastic Stretch Cavity Building” System in Endoscopic Thyroidectomy of Giant Thyroid Tumors
Source: Front Oncol. 2022 May 27;12:871594. doi: 10.3389/fonc.2022.871594 (PMC9186059; doi:10.3389/fonc.2022.871594)

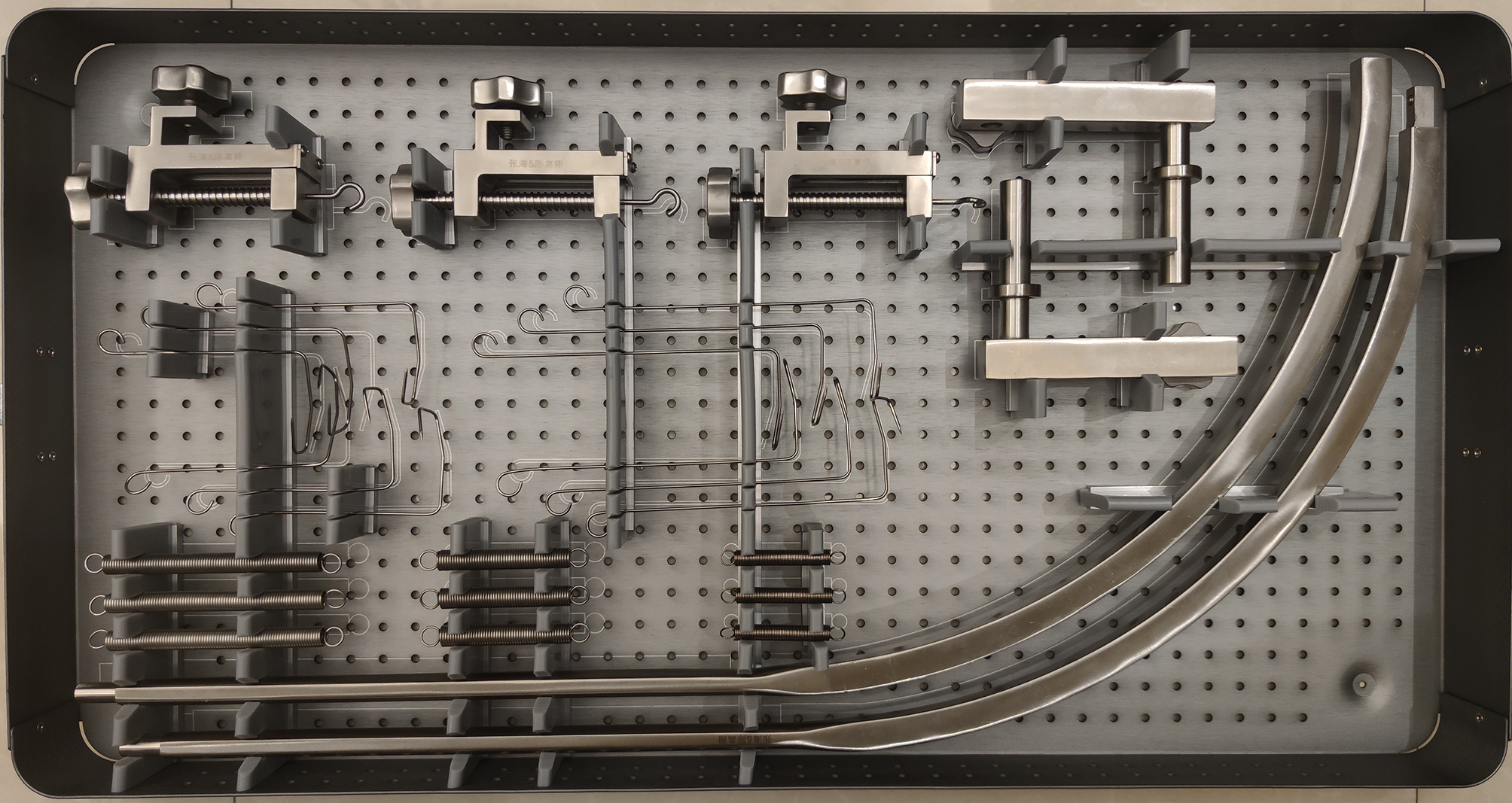

Supplement: Supplementary Figure 1 — The components of the “elastic stretch cavity builder”. [file Image_1.tif]
